# SFRP1 induces a stem cell phenotype in prostate cancer cells

**DOI:** 10.3389/fcell.2023.1096923

**Published:** 2023-03-09

**Authors:** Alberto Losada-García, Iván Salido-Guadarrama, Sergio Alberto Cortes-Ramirez, Marian Cruz-Burgos, Miguel Morales-Pacheco, Karla Vazquez-Santillan, Griselda Rodriguez-Martinez, Imelda González-Ramírez, Vanessa Gonzalez-Covarrubias, Carlos Perez-Plascencia, Mauricio Rodríguez-Dorantes

**Affiliations:** ^1^ Laboratorio de Oncogenomica, Instituto Nacional de Medicina Genomica, Mexico City, Mexico; ^2^ Departamento de Bioinformatìca y Análisis Estadísticos, Instituto Nacional de Perinatología Isidro Espinosa de los Reyes, Mexico City, Mexico; ^3^ Epigenetics Laboratory, Instituto Nacional de Medicina Genomica, Mexico City, Mexico; ^4^ Departamento de Atención a la Salud, Universidad Autónoma Metropolitana-Xochimilco, Mexico City, Mexico; ^5^ Pharmacogenomics Laboratory, Instituto Nacional de Medicina Genómica (INMEGEN), Mexico City, Mexico; ^6^ Unidad de Genómica y Cáncer, Subdirección de Investigación Básica, INCan, SSA and Facultad de Estudios Superiores Iztacala, Universidad Nacional Autónoma de México, Mexico City, Mexico

**Keywords:** prostate cancer, cancer stem cells, sfrp1, Wnt pathway, CAFs, stromal signaling

## Abstract

Prostate cancer (PCa) ranks second in incidence and sixth in deaths globally. The treatment of patients with castration-resistant prostate cancer (CRPC) continues to be a significant clinical problem. Emerging evidence suggests that prostate cancer progression toward castration resistance is associated with paracrine signals from the stroma. SFRP1 is one of the extracellular proteins that modulate the WNT pathway, and it has been identified as a mediator of stromal epithelium communication. The WNT pathway is involved in processes such as cell proliferation, differentiation, cell anchoring, apoptosis, and cell cycle regulation as well as the regulation of stem cell populations in the prostatic epithelium. In the present study, we explored the role of exogenous SFRP1 on the stem cell phenotype in prostate cancer. The results reveal that cancer stem cell markers are significantly increased by exogenous SFRP1 treatments, as well as the downstream target genes of the Wnt/-catenin pathway. The pluripotent transcription factors SOX2, NANOG, and OCT4 were also up-regulated. Furthermore, SFRP1 promoted prostate cancer stem cell (PCSC) properties *in vitro*, including tumorsphere formation, migration, bicalutamide resistance, and decreased apoptosis. Taken together, our results indicate that SFRP1 participates in the paracrine signaling of epithelial cells, influencing them and positively regulating the stem cell phenotype through deregulation of the WNT/β-catenin pathway, which could contribute to disease progression and therapeutic failure. This research increases our molecular understanding of how CRPC progresses, which could help us find new ways to diagnose and treat the disease.

## Introduction

Prostate cancer ranks second in incidences and sixth in deaths globally (Ferlay et al., 2021). It is well known that androgens are actively involved in the development of prostate carcinogenesis and disease progression, therefore the main clinical strategy is androgen deprivation therapy (ADT) either surgically or pharmacologically through drugs that block the androgen pathway. This androgen blockade is currently the indicated treatment for patients with advanced or metastatic prostate cancer; however, even initially good outcome prostate cancer patients usually progress to an advanced disease stage called androgen-independent cancer or castration-resistant prostate cancer (CRPC) (Watson et al., 2015; Pagliarulo, 2018). It has been reported that prostate cancer progressing to castration resistance may be associated with paracrine signals from the stroma mainly from cancer-associated fibroblasts (CAFs) (Bonollo et al., 2020; Lee et al., 2014). These stromal cells can be identified by specific markers including fibroblast activation protein (FAP), *a*-smooth muscle actin (ACTA2), fibroblast-specific protein one and CD90 (True et al., 2010; Wu et al., 2021; Remsing Rix et al., 2022). Stromal cells adjacent to prostatic adenocarcinomas are phenotypically different from stromal cells in disease-free areas and may influence tumor cell proliferation through an abnormal communication between reactive stroma and tumor epithelium. This may lead to the stimulation of tumor cell proliferation as well as the maintenance of the niche that supports prostate cancer stem cells (PCSCs) (Barron & Rowley, 2012; Loh & Ma, 2021). It has been reported that one of the mediators of stromal epithelial signaling is the SFRP1 protein which mediates stromal epithelial communication in prostate cancer (Joesting et al., 2005). SFRP1 is one of the extracellular proteins that can modulate the WNT pathway by interacting with both Wnt proteins and frizzled receptors through its cysteine-rich amino-terminal domain (CRD), also present in frizzled receptors (FZD) and a C-terminal domain that shares similarity with the axon guidance protein netrin (NTR) (Kawano & Kypta, 2003; Bovolenta et al., 2008). The SFRP family of proteins comprises five members (SFRP1-SFRP5) that are traditionally inhibitors of the WNT pathway. Accumulating evidence has shown that SFRPs exhibit dual roles since they can inhibit or activate the canonical WNT pathway, depending on the cellular context, concentration and FZD expression (Üren et al., 2000; Xavier et al., 2014; Liang et al., 2019). The WNT pathway is involved in processes such as cell proliferation, differentiation, cell anchoring, apoptosis and cell cycle regulation among others as well as the regulation of stem cell populations in the prostatic epithelium. Activation of WNT-β-catenin has been shown to promote prostate cancer stem cell expansion. There is also evidence that WNT-β-catenin pathway activity regulates prostate cancer stem cell self-renewal independently of androgen receptor activity (Bisson & Prowse, 2009; Murillo-Garzón & Kypta, 2017; Zhang et al., 2017). PCSCs exhibit self-renewal capacity, can produce different lineages in tumors, are responsible for therapeutic failure and promote metastasis to different organs showing great capacity for tumorigenicity *in vivo*. PCSCs have been identified in malignant prostate cancer tumors expressing markers: Integrin α2β1hi, CD133+ , and CD44^+^ (Patrawala et al., 2007; Pellacani et al., 2013; Packer & Maitland, 2016). In the present study, we explored the role of exogenous SFRP1 on the regulation of stem cell phenotype in prostate cancer.

## Materials and methods

### Transcriptome data retrieval and processing

Prostate adenocarcinoma RNA-Seq data from The Cancer Genome Atlas (https://cancergenome.nih.gov/) (TCGA-PRAD) were retrieved using the Xena Browser (https://xena.ucsc.edu/). Specifically, we selected the available gene count data derived from HTSeq software. RNA-Seq raw counts data were preprocessed as follows. In order to exclude low expressed genes that are not likely biologically relevant nor can be assessed for differential expression, only genes with at least 10 counts per million (cpm) in at least 70% of samples were retained. The filtered data were normalized using the cpm function with TMM normalization factor in EdgeR (Robinson, McCarthy, & Smyth, 2010) and log2 transformed.

### Stratification of TCGA-PRAD cases

Consensus clustering analysis based on the normalized expression of FAP and ACTA2 genes across samples was performed using the MC3 algorithm (John et al., 2020), with 25 iterations of resampling and a maximum of 10 partitions. Three different clustering algorithms were run (i.e., “pam,” “km,” “hc”). The most stable clustering solution was selected based on the Relative Cluster Stability Index (RCSI) computed by the algorithm. Subsequently, the EdgeR algorithm was used to perform a differential expression analysis between gene expression profiles of previously defined clusters.

### Cell signature enrichment analysis

To infer the cell admixture composition of PRAD samples, a deconvolution analysis was performed using the Xcell algorithm (Aran et al., 2017), which implements a gene-signature based method and includes signatures for 64 human immune and stromal cell types derived from expression profiles of thousands of pure cell types from different sources. Next, in order to discover differential enrichment of cell types between clusters, we performed a two-sided *t*-test, making pairwise comparisons of enrichment scores for each cell type of all samples, between clusters. The differences in enrichment scores were corrected for multiple hypothesis testing through the FDR method. An FDR <0.05 was the cutoff employed for significant differences in cell type enrichment.

### Gene enrichment analysis

Gene enrichment analysis was performed for differentially expressed genes between clusters using the Enrichr (https://maayanlab.cloud/Enrichr/). Genes were divided into upregulated and downregulated in each comparison and enrichment of “KEGG_2019_Human” terms was computed for each gene list. Significantly enriched terms were selected with an adjusted *p*-value <0.01.

### Cell culture

CAFs, LNCaP, and DU145 cell lines were purchased from ATCC (Manassas, United States). CAFs cells were grown in EMEM (ATCC 30-2003) supplemented with fetal bovine serum (FBS) (BioWest, South American origin) at 10%; 1% of sodium bicarbonate 7.5% solution (HycloneSH30033.01); puromycin (Gibco cat. A11138-03) for a final concentration of 1 μg/mL, in 37°C and 5% CO_2_ atmosphere. LNCaP cells were grown in RPMI 1640 medium (Sigma-Aldrich, St. Louis, MO, United States) supplemented with fetal bovine serum (FBS) (BioWest, South American origin), at 10% in 37°C and 5% CO_2_ atmosphere. DU145 cells were grown in DMEM 1640 medium (Sigma-Aldrich, St. Louis, MO, United States) supplemented with fetal bovine serum (FBS) (BioWest, South American origin), at 10%in 37°C and 5% CO_2_ atmosphere. When cells were treated with SFRP1 or bicalutamide, RPMI, or DMEM, medium without phenol red (Sigma) supplemented with 10% of charcoal-stripped FBS (Gibco cat.12676-029) was used.

### Treatments

To avoid hormone interference, all treatments were conducted in a medium containing fetal bovine serum, charcoal stripped (Gibco™, cat. 12676029). The treatments were performed with SFRP1 protein (R&D cat.5396-SF-025) at 0.1 nM with PBS carrier buffer as vehicle. The WNT/β-catenin pathway inhibitor ICRT14 (Toronto Research Chemical Canada I163900) at 12.9 μM in DMSO as a carrier buffer was employed (Trujano-Camacho et al., 2021). In drug resistance and apoptosis experiments, 50 and 25 μM concentrations in DMSO as carrier buffer of bicalutamide (Sigma-Aldrich cat. B9061) were used. The vehicle in control conditions (CTRL) was considered as PBS for SFRP1 and DMSO for Bicalutamide and ICRT14.

### RT-qPCR

Cells were plated in 25 cm2-angled flasks at 1 × 10^6 cells of confluence, 48 h after treatments, cells were scraped from flasks to extract RNA; the extraction was performed with RNAeasy kit (QUIAGEN, Hilden Germany) according to manufacturer’s instructions. Next cDNA was obtained by retro-transcription assay using Revert Aid Synthesis Kit (Thermofisher, United States). For RT-PCR assays, taqman probes used for: (Hs00610060_m1) SFRP1; (Hs00277039_m1) CCND1; (Hs03986777_s1) FZD4; (Hs02387400_g1) NANOG; (Hs99999903_m1) ACTB; (Hs00153408_m1) MYC; (Hs04260367_gH) POU5F1; (Hs00610344_m1) AXIN2; (Hs00602736_s1) SOX2; (Hs99999901_s1) 18S, and (Hs01547250_m1) LEF1 genes were purchased from Thermofisher (Massachusetts, United States).

### Western blotting

Cell protein extraction was performed with RIPA buffer (R0278 Sigma) according to the manufacturer’s protocol. For the proteins present in the conditioned media of CAFs; the cells were incubated with DMEM red phenol and serum-free for 48 h. CAFs, LNCaP, and DU145 conditioned medium was collected at 48 h post confluence and filtered with 0.2 μm filter to eliminate any detached cell. Then, proteins in conditioned media were precipitated as described (Koontz, 2014). Briefly, 100 μL of 100% TCA (Trichloroacetic Acid) was added to 1 mL of protein sample. The protein was then placed on ice for 60 min to precipitate. The samples were centrifuged at 10,000 G for 15 min at 4°C, followed by two washes with 500 μL of ice-cold acetone. After drying the pellet, it was resuspended in 100 μL of Ripa buffer. Proteins were quantified with DC Protein assay Reagent (BIO-RAD cat. 5000116). Western blotting assays were performed following the canonical steps. Proteins were run on 10% polyacrylamide gels and electrophoresis was carried out at 100 V during 2 h. A semi-dry system was used for transference to PVDF membrane at 20 V for 45 min. Next, we blocked the membrane with low fat milk at 5% in TBST overnight. After washes, primary antibodies were added in 5% low fat milk overnight in a shaker. Next day, the membrane was incubated with secondary antibodies in 5% low fat milk for 2 h. Membrane photos were taken after incubation with the HRP system Luminata Forte (Merck, Darmstadt, Germany) for 4 min. The antibodies used were: Anti SFRP1 ab267466; anti-Nanog ab21624; anti-Oct4 ab18976; anti-Sox2 ab97959; anti-Axin2 ab109307; anti-MYCN ab24193; anti-Beta actin ab8227; anti Lamin B1 ab16048; anti *a*-Tubulin 11H10; and anti-rabbit secondary antibody (HRP) ab205718.

### *In vitro* extreme limiting dilution analysis (ELDA)

The following amounts of cancer cells were plated in a 24-well ultra-low attachment plate: 4, 12, 36, 108, and 324 cells per well. Cells were treated with SFRP1 or vehicle, with six wells per dilution for LNCaP and DU145 cell lines, and then incubated for 7 days at 37°C under spheroid-forming conditions. Using visual inspection, the tumorspheres that eventually formed in each well were determined. For each dilution series, the number of wells exhibiting sphere formation on day 8 was recorded. The Walter and Eliza Hall Institute of Medical Research was used to calculate the frequency of cancer stem cells (http://bioinf.wehi.edu.au/software/elda/index.html).

### Flow cytometry analysis

For analysis of CD44+/CD133+ cells, LNCAP and DU145 tumorspheres were dissociated into single cells with Acutasse (Sigma-Aldrich cat A 6954) resuspended in 100 μL PBS buffer containing 10% BSA, and incubated with anti-CD44-FITC (MiltenyiBiotec) and anti-CD133-PE (MiltenyiBiotec) antibodies for 60 min on ice. CD44^+^ and CD133+ subpopulations were analyzed using flow cytometry (BD Biosciences, San Diego, CA, United States). For the apoptosis assay, cells were exposed to DMSO or bicalutamide 25 μM for 48 h. After washing with PBS buffer, cells were collected and incubated with FITC-conjugated Annexin V and propidium iodide (PI) according to the protocol of the FITC Annexin V/Dead Cell Apoptosis KIT (Life technologies cat. V13242). Apoptosis was analyzed by flow cytometry.

### Proliferation and cytotoxicity assays

LNCaP and DU145 cells were plated in 48-well plates at 1 × 1^4 cells per well. After adhering overnight, the cells were treated with bicalutamide (50 μM), bicalutamide plus SFRP1 or vehicle (DMSO) for 72 h. Cell viability was determined after treatment using the MTT assay kit (ab211091). For proliferation curve LNCaP and DU145 cells were plated in 48-well plates at 1 × 1^4 cells per well. After adhering overnight, the cells were treated with bicalutamide (50 μM), bicalutamide plus SFRP1 or vehicle (DMSO) for 96 h. Cell proliferation was determined every 24 h using the MTT assay kit (ab211091) following the manufacturer’s guidelines Briefly, 20 μL of MTT reagent was added per well, and cells were incubated at 37°C and 5% CO2 for 3 h. Next, 150 μL of MTT solvent was added to solubilize the formazan crystals. Absorbance was read at 590 nm wavelengths in both cell lines.

### Tumorsphere formation assay

The assay was performed as previously described in (Rodríguez-Dorantes et al., 2021). Briefly, 1,000 cells/mL of the LNCaP or DU145 cell lines were plated in 6-well ultra-low adherence plates (Corning 3,471) containing DMEM-F12 media supplemented with 20 ng/mL EGF, 20 ng/mL bFGF, ×1 B27, and 4 g/mL Insulin. Every 48 h, cells were treated with SFRP1 or a vehicle (PBS); spheroids were collected 14 days later for measuring, counting, and use in other assays.

### TOP/FOP flash assay

To determine the activity of the Wnt/β-catenin pathway, TOP/FOP flash assay (TCF Reporter Plasmid Kit Merck Millipore) was performed as previously described in (Trujano-Camacho et al., 2021). Briefly, 10 × 10^5 cells were seeded in a 6-well plate and co-transfected with 2.5 μg of TOP and FOP plasmids using Lipofectamine 2,000 transfection agent (Invitrogen). Cells were incubated with SFRP1 or vehicle. After 48 h, the cells of each group were collected, and then the activity of Wnt/β-catenin signaling pathway was measured by Dual-Luciferase Reporter Assay Kit (Promega) in GloMax^®^ 96 Microplate Luminometer (Promega; Madison, WI, United States).

### Colony formation assay

In 12-well plates, 500 cells were seeded and treated every 48 h with SFRP1 or vehicle. Two weeks after the colonies were plated, they were dyed with 0.1% crystal violet. The stained colonies were photographed and analyzed using ImageJ software.

### Wound healing and matrigel invasion assays

For wound healing assays, cells were plated in a 6-well plate and kept there until they reached 90% confluence. Then, a 200 μL pipette tip was used to scratch a cell monolayer. After washing the cells with PBS, the cells were grown in culture for another 48 h. At 0 h and 48 h, the width of the wound was measured. Cell migration was represented by the percentage of wound closure. For transwell assay, Corning^®^ BioCoat™ Matrigel^®^ Invasion Chamber (Cat. 354480) was used. The assay was performed following the manufacturer’s instructions. Briefly, the matrigel inserts were rehydrated using a medium free of SFB. After rehydration, 2.5 × 10^4 cells treated with SFRP or vehicle were seeded into the inserts, and a 10% SFB supplemented medium was utilized as a chemoattractant in the bottom half of the plate. The cells were cultured for 22 h at 37**°**C and 5% CO_2_ in a humidified tissue culture incubator. After incubation, non-invading cells were removed from the upper surface of the membrane by “scrubbing.” The inserts were rinsed twice with PBS and fixed for 2 min in methanol. The cells on the membrane’s lower surface were stained with 0.1% crystal violet, washed, and then were photographed and analyzed using ImageJ software.

### Statistical analysis

All statistical analyses were performed with Prism 8.0 software (GraphPad Software Inc., United States). The data are presented as the means ± SEM of three independent experiments. The Student's *t*-test was performed to analyze differences between the two groups, and ANOVA was performed to analyze differences among more than two groups. A *p*-value <0.05 was considered to be statistically significant.

## Results

### SFRP1 expression levels are associated with higher amount of CAFs

**FIGURE 1:**
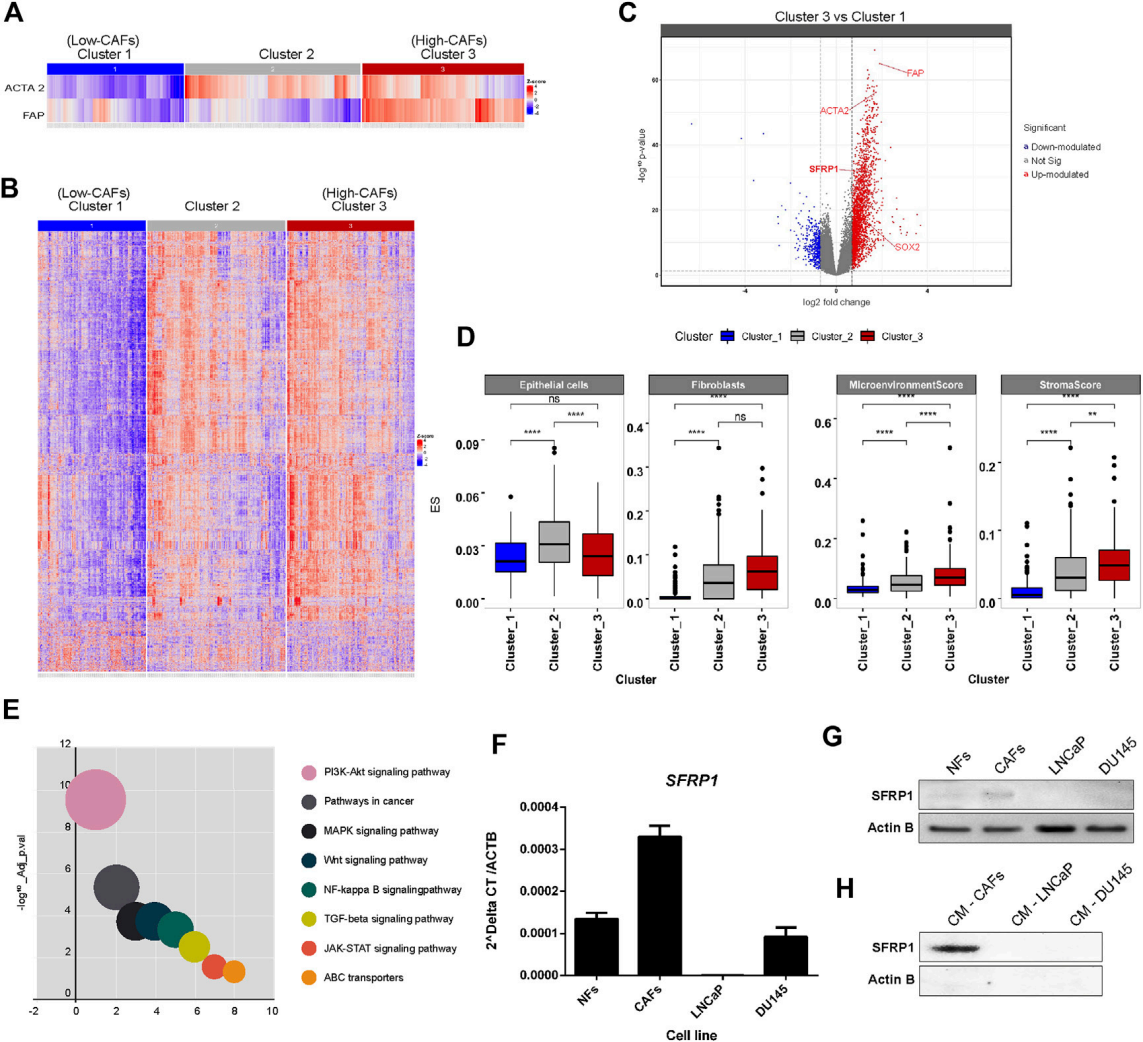
**(A)** Stratification of PRAD samples. Samples were clustered based on the expression of FAD and ACTA2 genes. A 3-cluster configuration was obtained with partition around medoid (PAM) algorithm from the Consensus Clustering Analysis. Heatmap shows the level of expression of both genes across samples in clusters. **(B)** Profile of differentially expressed genes between clusters. The heatmap shows the expression of differentially expressed genes between clusters of samples defined. Significant differences in gene expression were assessed and determined with a Fold-change >2 and adjusted *p*-value <0.05. **(C)** Volcano plot for differentially expressed genes between clusters, highlighting SFRP1 and SOX2 overexpression. **(D)** Differences in cell type enrichment between clusters. Comparison between the enrichment scores distributions of selected cell types are shown in the box and whiskers plot. Significant differences were determined with a two-sided *t*-test. Asterisk indicates differences with a *p*-value <0.05. **(E)** Significantly enriched KEGG terms in differentially expressed genes between cluster_3 and cluster_1 (relevant pathways in cancer). **(F)** qPCR for SFRP1 transcript in normal fibroblasts (NFs), cancer-associated fibroblasts (CAFs), LNCaP, and DU145 cell lines (* *p*-value<0.05). **(G)** Western blot for SFRP1 protein in NFs, CAFs, LNCaP, and DU145 cell lines. **(H)** CM-CAFs; CM-LNCaP, and CM-Du145 represent SFRP1 in conditioned medium of each cell line.

Initially we performed a bioinformatic study to determine if a higher amount of CAFs corresponds to a higher expression of SFRP1. As we mentioned before, CAFs are characterized by FAP and ACTA2 expression. For which, We conducted an unsupervised analysis using a Consensus clustering algorithm (John et al., 2020) based on the expression of FAP and ACTA2 from TCGA-PRAD RNA-seq data. The partition around medoids (PAM) allowed us to achieve the most stable clustering solution, with RCSI = 2.017 and *p*-value = 0.04 (Supplementary Figure 1), in which we defined three groups of samples, categorized according to their FAP and ACTA2 expression signature, Cluster one having a low FAP and ACTA2, Cluster 2 with a low FAP and high ACTA2 and Cluster 3, which has high FAP and ACTA2 levels (Figure 1A). To further explore the biological and molecular consequences of altered expression of FAP and ACTA2 in PCa, then, we focused on global gene expression discrepancies between Cluster three and Cluster 1. We identified a total of 2,700 differentially expressed genes (i.e., fold-change two and FDR <0.05) (Figure 1B), out of which 2,400 were upregulated while 300 genes were downregulated in Cluster_3 samples, with high FAP and ACTA2 expression (Figure 1C). Interestingly, we discovered that SFRP1 and SOX2 were among the upregulated genes. Given that our analysis was conducted on gene expression profiles obtained from RNA-seq bulk experiments, we pondered whether there might be a significant enrichment in gene expression signatures of cell types indicating a higher abundance of CAF in tumour samples with overexpression of FAP and ACTA2 in Cluster 3. In order to achieve this, we performed a digital cellular deconvolution analysis on the same gene expression data and compared the cell enrichment scores for 64 distinct cell types between clusters. We observed a considerable increase in fibroblasts in Cluster three samples (Figure 1D). In addition, we observed considerably higher StromaScore and Microenvironment Score values, which are associated with fibroblast and endothelial cell infiltration (Aran et al., 2017). Due to the importance of CAFs in cancer manifestation, we examined the probable mechanisms by which CAFs influence the growth and development of tumours, therefore, to understand the series of biological processes associated with FAP, ACTA, and SFRP1 over-expression and higher fibroblast and stromal scores. We analyzed the enrichment of KEGG pathways terms in the list of differentially expressed genes in Cluster_3. ABC transporters, JAK-STAT, TGF-beta, MAPK, and WNT signaling were found among the enriched terms (Figure 1E). The complete list of pathways is shown in Supplementary Table 1.

It has previously been documented that normal fibroblasts and CAFs express SFRP1 differently. SFRP1 is overexpressed in cancer-associated fibroblasts (CAFs) compared to normal fibroblasts (Joesting et al., 2005; Clark et al., 2013; Kato et al., 2020). Similarly, in colon cancer, a subpopulation of CAFs that overexpress Sfrp1/2 have been identified, promoting greater rates of malignancy in intestinal tumors. This subgroup is regulated by PKCζ, which was revealed to be downregulated in CAFs. The loss of expression of PKCζ is required for the induction of SOX2, which binds directly to the SFRP1/2 promoter, enhancing its expression (Kasashima et al., 2021). This is consistent with our analysis of TCGA data, which revealed a greater expression of SOX2 and SFRP1 in CAF-enriched samples. In addition we performed RT-qPCR and western blot analysis to compare SFRP1 expression between normal fibroblasts (NF), cancer-associated fibroblasts (CAFs), LNCaP, and DU-145 prostate cancer cell lines. Our results showed that SFRP1 levels were higher in CAFs than in NF; LNCaP and DU145 cell lines, as shown in Figure 1F. Similarly, the Western blot analysis verifies these data (Figure 1G). Moreover, it demonstrates that SFRP1 is present in the conditioned medium of CAFs but undetectable in the conditioned media of LNCaP and DU145 cell lines (Figure 1H). Together, these data and the bioinformatics analysis are consistent with previously reported data, indicating that SFRP1 participates in stromal-epithelial signaling, which opens the door for examining the effect of exogenous SFRP1 on PCSC properties and the canonical WNT pathway.

### SFRP1 enhances the expression of cancer stem cell-associated genes

**FIGURE 2:**
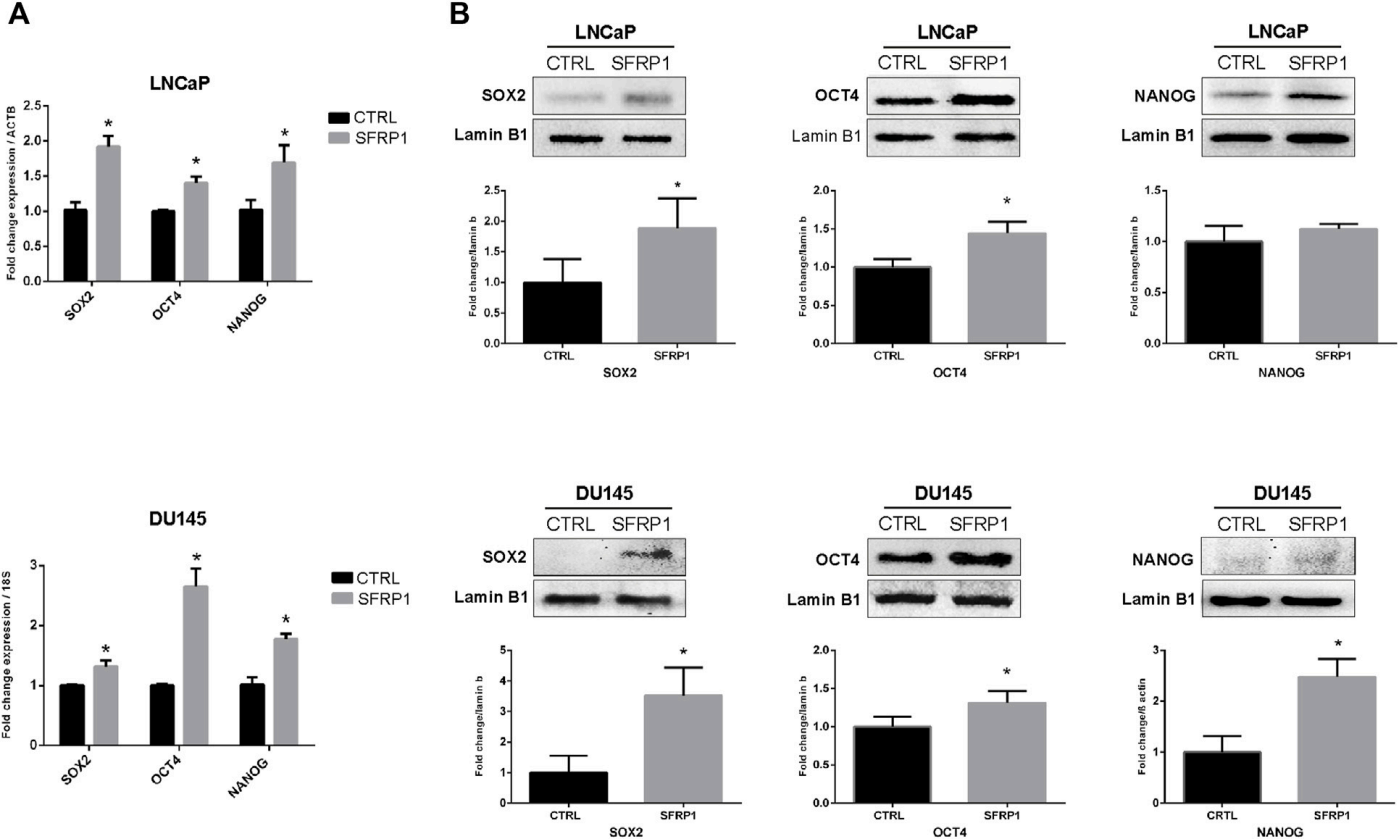
**(A)** LNCaP and DU145 cells were incubated for 48 h with SFRP1 or vehicle. Expression levels of SOX2, OCT4, and NANOG were determined by qPCR. Data shows relative mRNA expression; *ß*-actin (LNCaP) and 18S (DU145) were used as housekeeping gene (*p < 0.05). **(B)** Protein levels were determined by Western blot, lamin b1 was used as loading control, (*p < 0.05).

An RT-qPCR assay confirmed that the cancer stem-associated transcripts SOX2, OCT4, and NANOG were substantially upregulated by SFRP1 exposure, in both LNCaP and DU145 (Figure 2A). Likewise, in the western blot assay observed an increase in SOX2, and OCT4, however not in NANOG for LNCaP cell line (Figure 2B up). When DU145 was treated with exogenous SFRP1, the similar responses were observed, including an increase in SOX2, OCT4, and NANOG (Figure 2B down). These results showed that SFRP1 could upregulate the mRNA expression of genes linked with cancer stem cells, supporting our prior findings.

### SFRP1 promotes cancer stem cell like population

**FIGURE 3:**
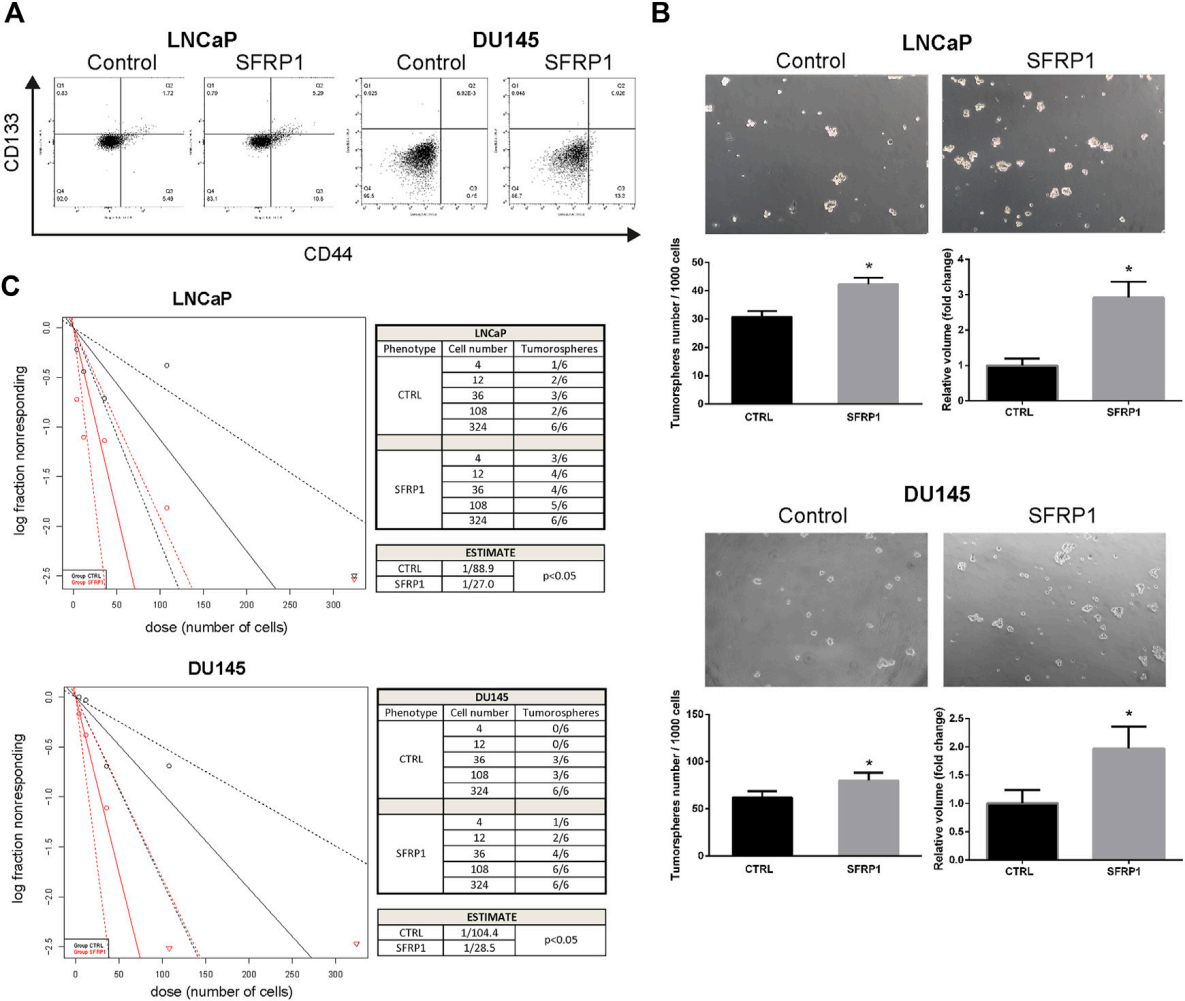
**(A)** Flow cytometry analysis of the CD44+/CD133+ subpopulation in cells treated with SFRP1 or vehicle. **(B)** Representative images of tumorspheres formed in LNCaP and DU145 cells treated with SFRP1 or vehicle; tumorspheres were counted from 1,000 seeding cells, and the volume was calculated with the equation V = 4/3 πr³ (*p < 0.05). **(C)** The χ2 tests were calculated using ELDA software to evaluate the frequency of PCSCs. The number of cells plated is displayed against the log proportion of wells devoid of tumorspheres. The tables illustrates the estimated frequency of PCSCs.

**FIGURE 4:**
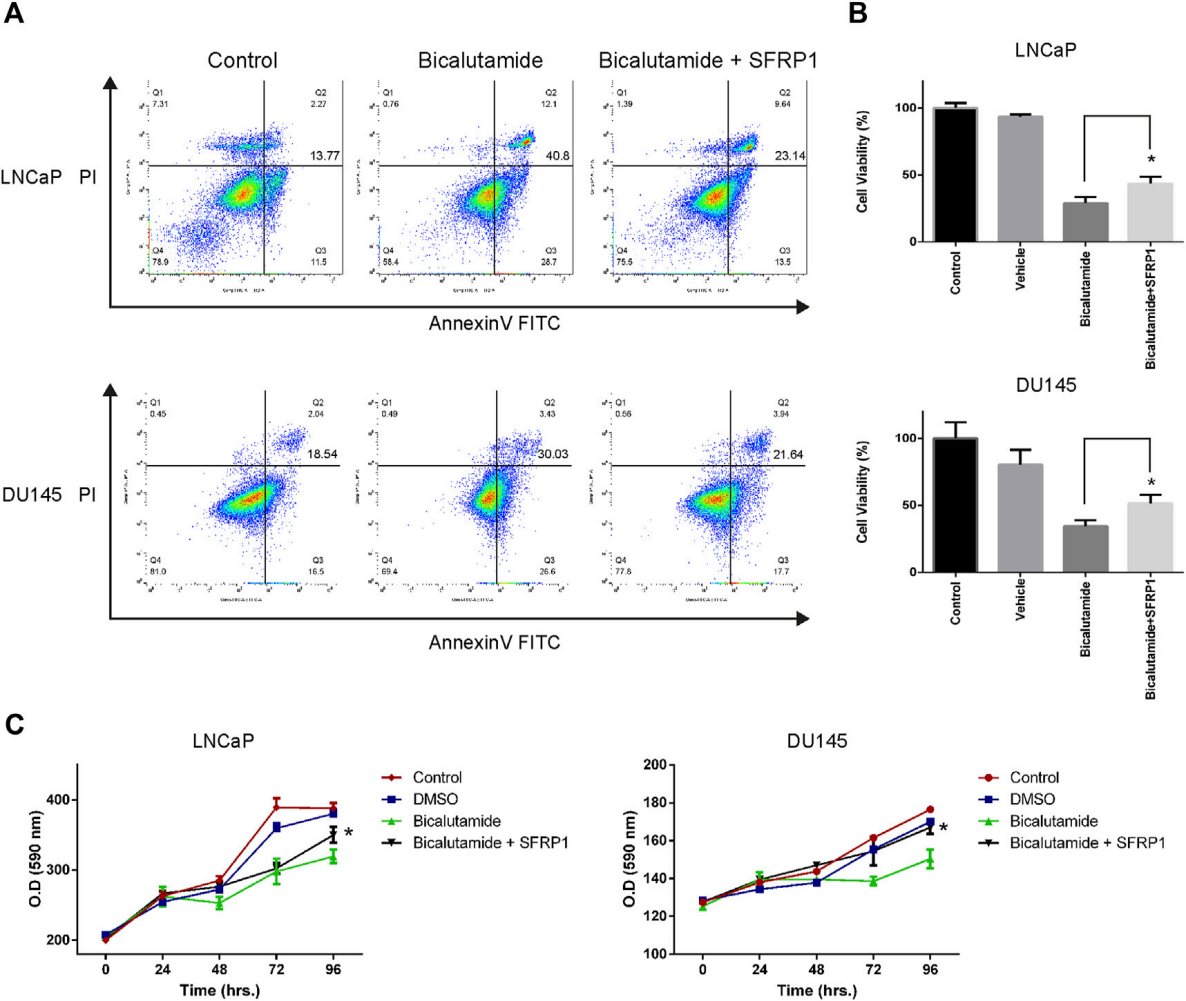
**(A)** Cell apoptosis of LNCaP and DU145 was detected by flow cytometry. Cells were treated with either SFRP1 or vehicle (CTRL); FITC-Annexin V coupled antibody and propidium iodide was used for the detection. **(B)** MTT assay was used to determine cytotoxicity. LNCaP and DU145 cells were treated for 72 h with bicalutamide (50 μM) or bicalutamide + SFRP1 or vehicle, (*p < 0.05). **(C)** Proliferation curve was perform using MTT assay kit. LNCaP and DU145 cells were treated for 24, 48, 72 and 96 h with: Bicalutamide (50 μM), bicalutamide + SFRP1 or vehicle, every 24 h the O. D (590 nm) was obtained (*p < 0.05).

We evaluated whether SFRP1 may modulate prostate cancer stem cell populations and features associated with stem cells in PCa cells. Using flow cytometry, we analyzed the expression of the previously described CD44 and CD133 markers (Packer & Maitland, 2016). As shown in Figure 3A, the treatment increase the amount of the CD44+/CD133+ populations suggesting an increase in the quantity of cancer stem cells. Since the formation of tumorspheres is essential for assessing PCSCs. We conducted a tumorosphere formation assay and an Extreme Limiting Dilution Analysis (ELDA) to validate previous findings. We demonstrate that treatment with SFRP1 increased both the number and size of tumorospheres in LNCaP and DU145 cell lines (Figure 3B). Additionally, in ELDA a rise in stem cell frequency was found, LNCaP CTRL 1/88.9; LNCaP SFRP1 1/27; DU145 CTRL 1/104.4; DU145 SFRP1 1/28.5 (Figure 3C). Based on the fact that the SFRP treatment impacts PCSC populations and they play an important role in the recurrence of CRPC, we aimed to see if SFRP1-treated cells had enhanced drug resistance to bicalutamide, a common medication used to treat prostate cancer. First, it was determined whether SFRP1 affected apoptosis in cells. The overall rate of apoptosis was found to be lower in the bicalutamide + SFRP1 group compared to the bicalutamide group (Figure 4A). In order to corroborate these results, we treated LNCaP and DU145 cell lines with either 50 μM of bicalutamide, or 50 μM of bicalutamide + SFRP1, or vehicle, and cell viability was evaluated at 72 h (Figure 4B). In addition, a proliferation curve was performed from 24 h to 96 h with the same treatments (Figure 4C). Our results demonstrate that cells treated with bicalutamide + SFRP1 had a significant increase in drug resistance and enhanced survival compared to the bicalutamide group. Taken together these findings indicate that SFRP1 exposure could increase the amount of PCSCs, hence increasing the number of self-renewing cells. They also demonstrate that SFRP1 is important for the survival of cancer stem cells and prostate cancer drug resistance.

### SFRP1 encourages cancer’s aggressive properties

**FIGURE 5:**
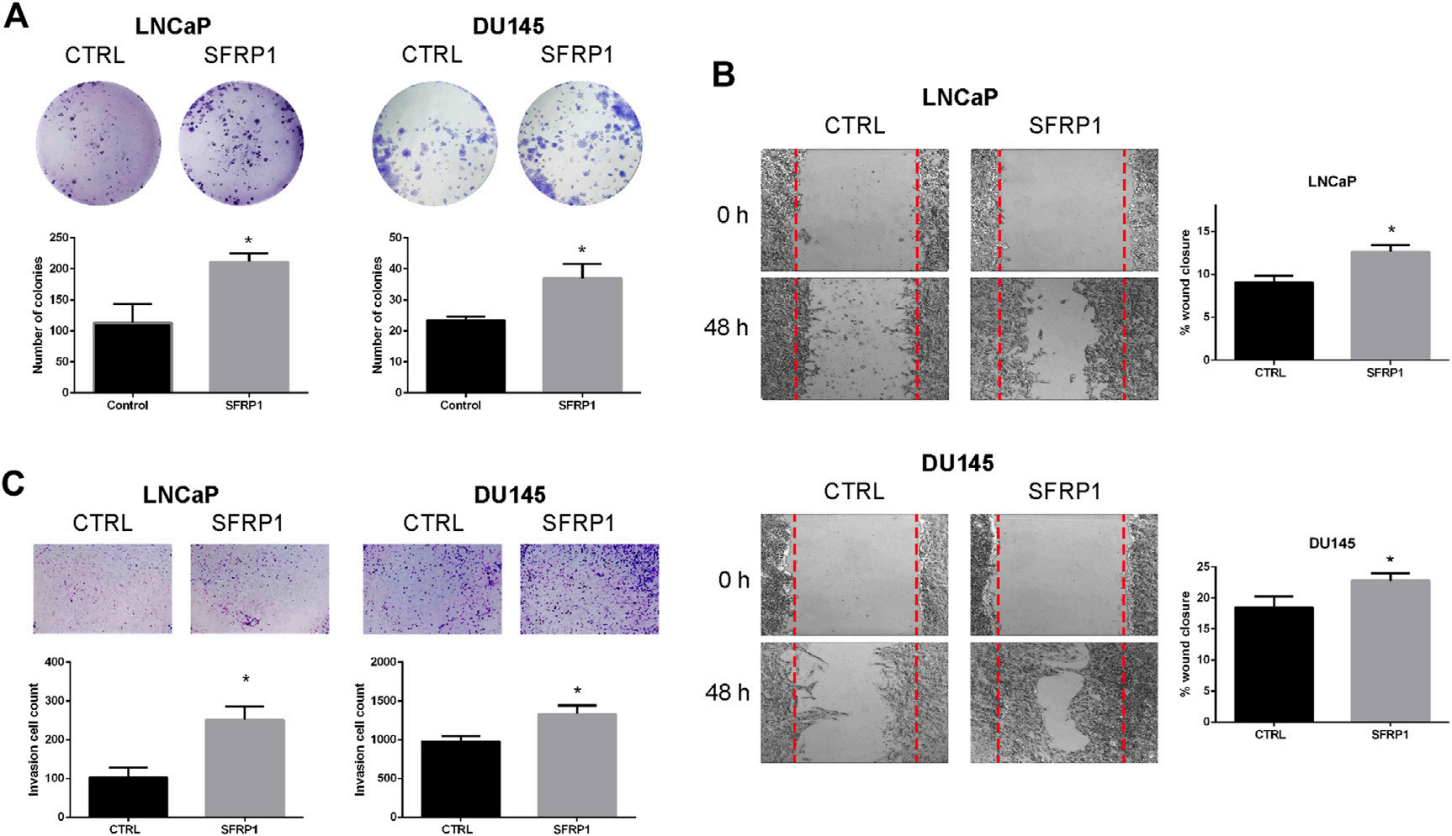
**(A)** The clonogenic ability of LNCaP and DU145 was analyzed. Cells were treated with SFRP1 or vehicle for 2 weeks, after incubation, cells were stained with crystal violet and analyzed using ImageJ software. **(B)** Cell migration of LNCaP and DU145 cells was detected by a wound healing assay. Cells were treated with either SFRP1 or vehicle for 48 h before their evaluation **(C)**. Cell invasion of LNCaP and DU145 cells was detected by the Transwell assay. Cells were treated with either SFRP1 or vehicle and the invading cells were counted (*p 0.05).

We analyzed migration, invasion, and colony formation to establish whether SFRP1 could boost cancer’s aggressive properties. We evaluate the clonogenic ability of LNCaP and DU145 cells with vehicle (CTRL) or SFRP1 treatment. Colony formation assays revealed that SFRP1 treatment increased the total number of colonies (defined as >20 μm) in LNCaP and DU145 cells by 86% and 58%, respectively (Figure 5A). A wound healing assay experiment was conducted to evaluate the effect of SFRP1 on PCa cell migration. As observed in Figure 5B, with SFRP1 treatments cell migration increased in both LNCaP and DU145 cell lines. Transwell invasion assay also significantly increased the number of cells that entered Matrigel, suggesting that SFRP1 promoted the stimulation of cell invasion characteristics related to PCa metastasis (Figure 5C). These findings demonstrate that SFRP1 plays an important role in PCa malignancy promotion.

### SFRP1 contributes WNT/β-catenin activation

**FIGURE 6:**
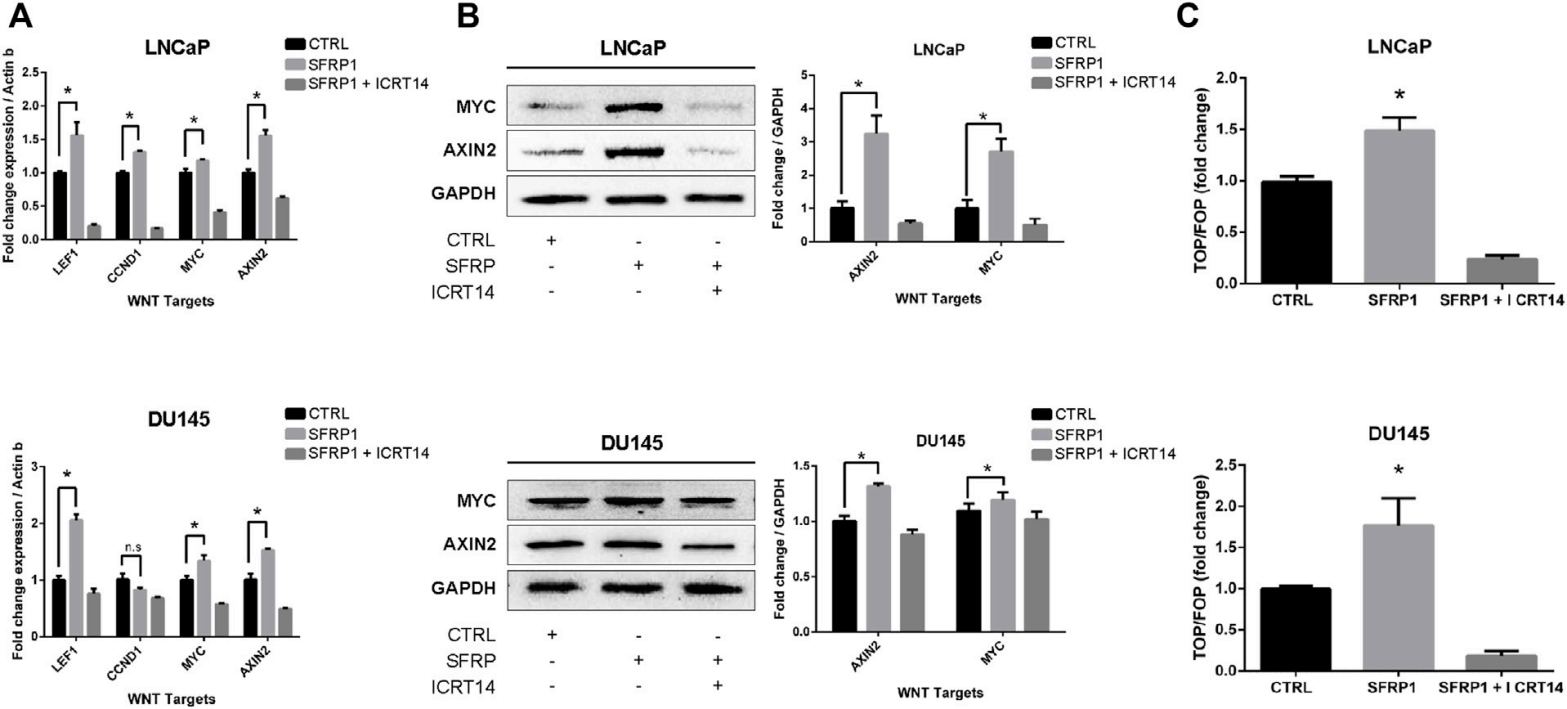
LNCaP and DU145 cell lines were treated with SFRP1; SFRP1 + ICRT14 or vehicle for 48 h. **(A)** The differential expression of WNT target genes was evaluated using qRT–PCR. Actin-b served as the normalization control. **(B)** Western blot was used to measure the protein levels of AXIN2 and MYC. The relative expression was quantified using ImageJ software and is displayed below the western blots (fold). **(C)** Luciferase reporter assay of *ß*-catenin-Tcf/Lef activity was used. LNCaP and DU145 cells were transfected with the Wnt signalling reporter (TOP flash or the control FOP flash). It shows the TOP/FOP fold change relative to control (*p < 0.05).

As noted previously, SFRP1 can have a dual role in WNT/β-catenin pathway signaling; therefore, to determine if SFRP1 promotes the activation of WNT/β-catenin the expression of target WNT genes AXIN2, NMYC, LEF, and CCND1 were evaluated with an RT-qPCR assay and western blot. As shown in Figure 6A (RT-qPCR), and 6B (Western blot assay), exposure to SFRP1 promotes the expression of genes associated to the activation of the WNT canonical pathway in both LNCaP and DU145 cells. To test if SFRP1 promotes the Wnt/β-catenin pathway in cell lines, we carried out a TOP-flash experiment, the standard assay for assessing Wnt/β-catenin pathway activity; LNCaP and DU145 were examined. Results indicated that the SFRP1-treated group exhibits increased TOP/FOP activity relative to the control group in both cell lines LNCaP and DU145 (Figure 6C). All of these findings suggest that SFRP1 enhances the activation of the WNT/β-catenin pathway in the context of our model.

## Discussion

CAFs are a crucial component of the tumour microenvironment and are frequently associated with metastasis and treatment resistance (Bhowmick et al., 2004; Kalluri, 2016). CAFs also encourage the progression of prostate cancer in surrounding epithelial cells (Olumi et al., 1999; Kato et al., 2020). CAFs signalling has been documented promote cancer cell growth, stemness, migration, and invasion (W.-J. Chen et al., 2014; X; Chen & Song, 2019). In this study we demonstrated that CAF-enriched samples (ACTA2 and FAP high) displayed an enrichment in fibroblast-associated molecular signatures as well as a decrease in epithelial cells, which was associated with an overexpression of SFRP1, corroborating the importance of SFRP1 in stromal-epithelial signalling in the tumour microenvironment and its relationship to advanced disease stages (Joesting et al., 2005; Wu et al., 2021). In many cases, castration-resistant prostate cancer is related with the induction of developmental programmes, stem cell-like phenotypes in response to treatment (Beltran et al., 2020). Our results showed that treatments with exogenous SFRP1 led to phenotypic redifferentiation toward a “stem-like” state. These treated cells showed a upregulation of CD133 and CD44; resistance to bicalutamide and exhibited overexpression of the transcription factors SOX2, NANOG, and OCT4. Both CD133 and CD44 have been identified as markers of prostate cancer stem cells (Packer & Maitland, 2016), and has recently been linked to drug resistance, since CD133 knockdown decreased the growth, proliferation, and migration of prostate cancer cells *in vitro* and enhanced their sensitivity to paclitaxel (Aghajani et al., 2020). Similarly, CD44 inhibition resulted in the suppression of migration and invasion of docetaxel-resistant PC/DX25 and DU/DX50 cells (Lai et al., 2019).

Castration-resistant prostate tumours can be reprogrammed into other cell lineages, hence acquiring phenotypic plasticity. Since there is no recognised treatment, this poses a significant challenge for clinical patient care (Blee & Huang, 2019). It has been identified that SFRP1 is overexpressed in the stromal cells of the prostate tumour. When stromal SFRP1 was added to a prostatic epithelial cell line, cell proliferation increased, and cell death decreased. This shows that prostate tumour stroma SFRP1 signalling transmits growth signals to adjacent epithelial cells (Joesting et al., 2005). However, authors did not evaluate the expression of stem cells markers or stem cells features. Self-renewal capacity, as determined *in vitro* by the ability of prostate cancer cells to create tumorspheres, is one of the most important properties of CSC (S Franco et al., 2016)**.** Our findings revealed that SFRP1 treatment increased expression of stem cells markers CD44, CD133 as well as the number of tumorspheres. The Extreme Limiting Dilution Analysis (ELDA) also revealed that the treatment with SFRP1 increased the number of stem cells frequency. Other investigations found that LNCaP spheres grown in serum-free media for 12 days contained more of the stem cell marker CD44 and were more resistant to apoptotic stimuli (Y. Wang et al., 2015). This 3D culture has been reported as a technique for enriching cancer stem cells and simulate tissue-like properties (Takagi et al., 2007; Fan et al., 2012). The results presented in this article prove that SFRP1 reduced the susceptibility of prostate cancer cells to bicalutamide. This suggests that this resistance may developed by an increase in cells that acquire PCSC characteristics after exposure to SFRP1.

SOX2, OCT4, and NANOG are the major transcriptional regulators that preserve the undifferentiated state of stem cells (Young, 2011; Kumar et al., 2012; Swain et al., 2020). Our work revealed that exposure to SFRP1 leads to an increase in the expression of SOX2, OCT4, and NANOG. The relationship between SOX2 expression and CSCs is well-established in a variety of cancer types, and SOX2 positive prostate cancer stem cells play an essential role in tumour development and therapeutic resistance (Vaddi et al., 2019). Also, an increase in the expression of SOX2 has been associated with alterations in several metabolic pathways, a shorter period until metastasis, and a shorter time for patients to live following biochemical recurrence (de Wet et al., 2022). Multiple studies have discovered that the master regulators of prostate cancer stem cells are associated with tumour progression. Patients with a poor prognosis had tumours with higher levels of the pluripotent markers OCT4 and SOX2, in addition to the polycomb complex protein Bmi-1 (Kerr & Hussain, 2014). In prostate cancer, rectal cancer, glioma, melanoma, medulloblastoma, acute myeloid leukaemia and other cancer types, high OCT4 mRNA or protein expression was related with poor clinical outcomes (Mohiuddin et al., 2020). Furthermore, it has been demonstrated that docetaxel and mitoxantrone resistant cell lines had elevated levels of POU5F1/OCT4 and a high tumorigenic potential in NOD/SCID mice (Linn et al., 2010). Similarly, NANOG has been established as a marker for cancer stem cells. Its overexpression in cell lines increased MCF-7 cell drug resistance, tumour regeneration in DU145 cells, and, most significantly, the growth of castration-resistant tumours in LNCaP cells. These pro-tumorigenic effects of NANOG were associated with significant molecular alterations, including the upregulation of markers such as CD133 and ALDH1 (Jeter et al., 2011; Vasefifar et al., 2022). All of these studies support our findings and demonstrate that an increase in the expression of these transcription factors constitutes a stem-cell phenotype, inducing treatment resistance, a high tumorigenic capability, and the progression of cancer.

**FIGURE 7:**
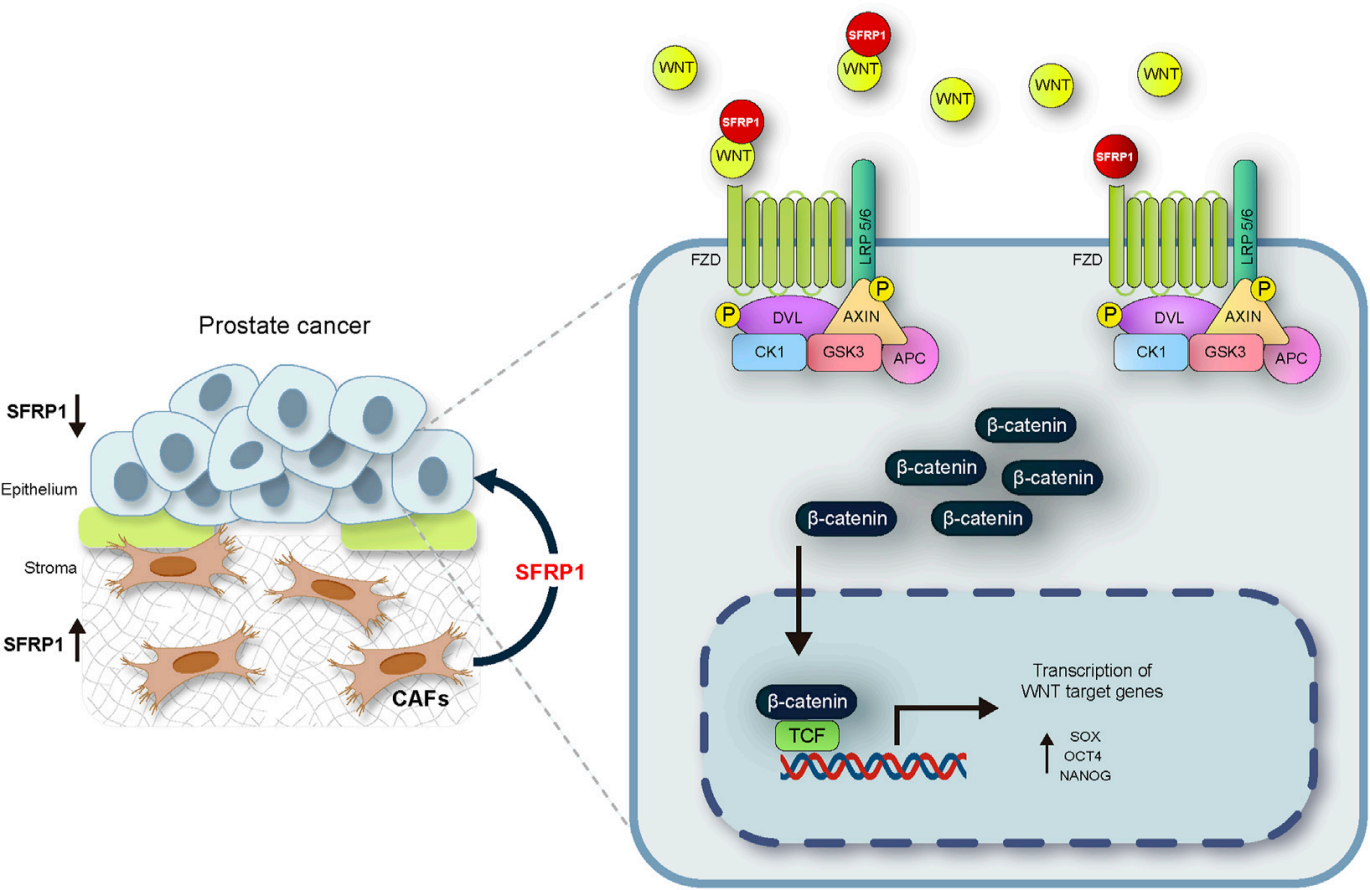
The suggested mechanism by which the loss of SFRP1 expression in epithelial cells linked with the stromal SFRP1 signalling axis promotes the stemness of prostate cancer cells and reduces their sensitivity to bicalutamide. SFRP1 induces higher SOX2, OCT4, and NANOG expression levels, all within the context of WNT/β-catenin pathway activation.

The mechanisms of prostate cancer initiation and metastasis have been intensively studied. There is evidence that PCSCs play essential functions in metastasis through partial EMT (Mei et al., 2019). Invasion and migration are two phenomena associated with cancer stem cells in a variety of cancer types. (Nam et al., 2015; Thomas et al., 2016; Harris & Kerr, 2017; Walcher et al., 2020). The expression of the cancer stem cell markers SOX2, OCT4, and NANOG also has been also associated to these features. (Dai et al., 2013; Siu et al., 2013; Novak et al., 2020). In our research we discovered that SFRP1 increased migration and invasion in LNCaP and DU145 cell lines while inhibiting apoptosis. In addition, we confirmed that cells treated with SFRP1 showed increased clonogenic potential. This is also consistent with other studies assessing the clonogenic potential and drug resistance of prostate cancer stem cells (Todaro et al., 2007; Rajendran & Jain, 2018; Lin et al., 2022). It has been reported that SFRP1 is downregulated in epithelial lines of prostate cancer by a mechanism that involves DNA hypermethylation and an increase in H3K27me3 signal in histones (García-tobilla et al., 2016). A negative association between SFRP1 expression and cancer progression has also been reported, showing that loss of SFRP1 expression is a promoter of theWnt/β-catenin pathway (Li et al., 2017; Song et al., 2018; Sunkara et al., 2020; Yu et al., 2022). However, these investigations did not examined the tumour microenvironment including the stromal signalling. In this investigation, we confirm that SFRP1 is suppressed in prostate cancer epithelial cell lines, and expressed in CAFs, we have also demonstrated that SFRP1 was secreted to the media by CAFs. This is the first study to demonstrate that exogenous SFRP1 signaling is implicated in the promotion of a stem cell phenotype in prostate cancer epithelial cells and this may be mediated by the activation of the WNT pathway. Our conclusion is supported by gathering four pieces of evidence. First, the bioinformatic analysis shows that the expression of SFRP1 is significantly elevated in CAF-rich samples (high FAP and ACTA2, cluster 3) in the TCGA data set. Second, SFRP1-treated cells overexpressed stem cell-associated markers, CD44, CD133, SOX2, NANOG, and OCT4, indicating the induction of the stem-cell phenotype. Third, we reported increased stem cell-related features including migration, invasion, colony formation, and sphere formation. Fourth, we demonstrated that cells treated with sfrp1 upregulate genes involved in the activation of the canonical WNT pathway. According to reports, the activation of the WNT/β-catenin pathway is closely associated with the progression and maintenance of stem cell self-renewal in numerous types of cancer (Holland et al., 2013; Murillo-Garzón & Kypta, 2017; S; Wang et al., 2014). Various studies have also shown that SFRP1 can promote the activation of the canonical WNT pathway (Üren et al., 2000; Xavier et al., 2014; Cruz-Hernández et al., 2020), Considering the results of this research and those described in the literature, we propose a possible mechanism by which the loss of SFRP1 expression in epithelial cells together with SFRP1-mediated stromal signalling may support the activation of the WNT/β-catenin pathway and contribute to the development of stem-like properties in prostate cancer (Figure 7). This report extends our understanding of the underlying molecular mechanisms behind how prostate cancer progresses into advanced states of disease. The complicated interaction between the stroma and epithelium in cancer requires additional research. This knowledge could help in the identification of new diagnostic and therapeutic targets and facilitate future investigation into the mechanisms regulating the progression to hormone-resistant prostate cancer.

## Data Availability

The datasets presented in this study can be found in online repositories. The names of the repository/repositories and accession number(s) can be found in the article/Supplementary Material.
