# Supplementary material for: SFRP1 induces a stem cell phenotype in prostate cancer cells
Source: Front Cell Dev Biol. 2023 Mar 9;11:1096923. doi: 10.3389/fcell.2023.1096923 (PMC10033548; doi:10.3389/fcell.2023.1096923)
Supplement: Supplementary file 3 [file Table1.DOCX]

Supplementary Material


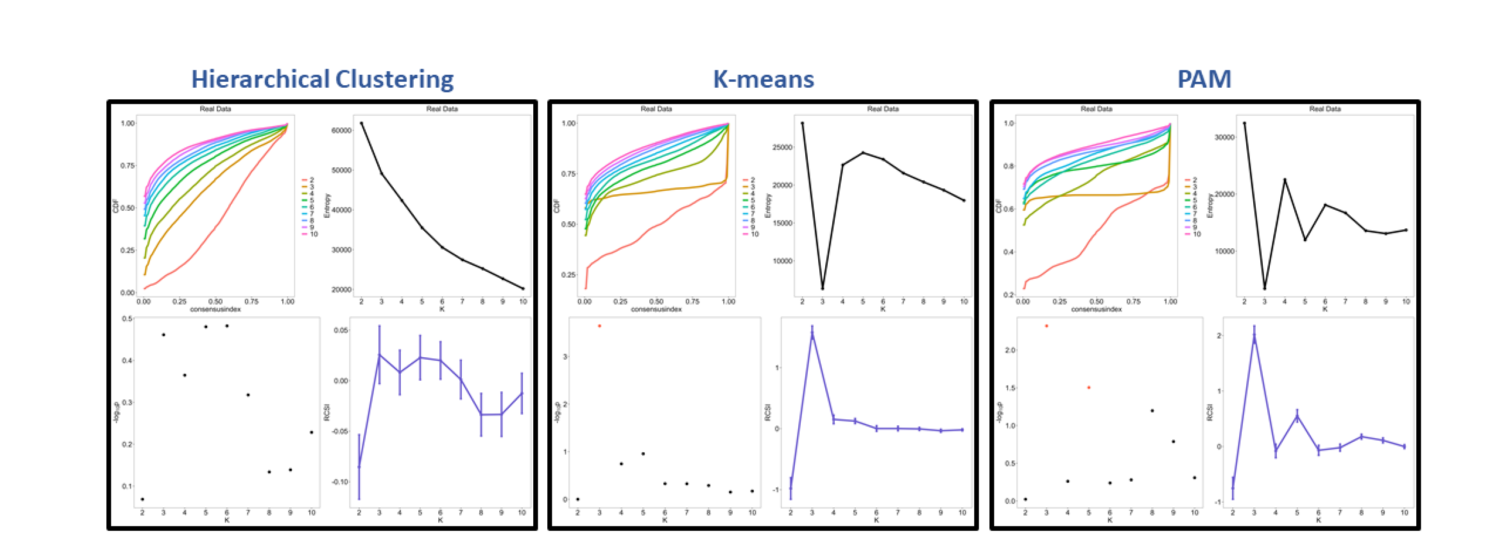


**Supplementary Figure 1.** Unsupervised analysis based on the expression of FAP and ACTA2 in TCGA-PRAD RNA-seq data using a consensus clustering algorithm It shows that the partition around medoids (PAM) is the most stable clustering option, with RCSI = 2.017 and p-value = 0.017.
